# Lithotomy

**Published:** 1841-08

**Authors:** 


### LITHOTOMY.

This operation was performed with complete success by Dr. Debow, of Hartsville, Tenn., on the first day of last May. The subject was a negro boy, belonging to Mr. Stubblefield, four years of age. The stone removed was as large as a pigeon's egg, and was of the fusible variety.

Dr. Debow is one of half a dozen young surgeons in Tennessee who have operated for stone in the bladder, within a few years, and with a success not surpassed by veteran surgeons. Of five and twenty such operations, performed upon patients of all ages, which have come to our knowledge, not one has had an unfavorable termination.

